# Sun-Stroke

**Published:** 1871-08

**Authors:** 


### SUN-STROKE

May be prevented by carrying in the crown of the hat a few green leaves, a silk handkerchief, or a dampened paper crumpled up. These serve to cool the air in the hat, and prevent the sun's rays from beating on the brain and disorganizing the blood in a way to produce gases, which distend the blood-vessels, thus causing a pressure on the brain, inducing loss of sense, as in apoplexy. A stroke of lightning acts in the same way. The speediest mode of cure, almost instantaneous, is to relieve the pressure in the blood-vessels by bleeding from both arms at the same time.

The next best plan is to place the person in a chair so as to favor the blood running downwards from the head; then envelop it in a bag of powdered ice, or clothes wrung out in ice-water, renewed every minute, in a way not to allow the water to fall on the clothing. Every few minutes give a hot drink of some kind. Any heat may cause sun-stroke.

